# RNA Sequencing of the Exercise Transcriptome in Equine Athletes

**DOI:** 10.1371/journal.pone.0083504

**Published:** 2013-12-31

**Authors:** Stefano Capomaccio, Nicola Vitulo, Andrea Verini-Supplizi, Gianni Barcaccia, Alessandro Albiero, Michela D'Angelo, Davide Campagna, Giorgio Valle, Michela Felicetti, Maurizio Silvestrelli, Katia Cappelli

**Affiliations:** 1 Department of Pathology, Diagnostic and Veterinary Clinic - Sport Horse Research Centre, University of Perugia, Perugia, Italy; 2 CRIBI, University of Padua, Complesso Vallisneri, Padova, Italy; 3 Laboratory of Genetic and Genomics, DAFNAE - University of Padova, Campus of Agripolis, Legnaro, Italy; Wageningen UR Livestock Research, The Netherlands

## Abstract

The horse is an optimal model organism for studying the genomic response to exercise-induced stress, due to its natural aptitude for athletic performance and the relative homogeneity of its genetic and environmental backgrounds. Here, we applied RNA-sequencing analysis through the use of SOLiD technology in an experimental framework centered on exercise-induced stress during endurance races in equine athletes. We monitored the transcriptional landscape by comparing gene expression levels between animals at rest and after competition. Overall, we observed a shift from coding to non-coding regions, suggesting that the stress response involves the differential expression of not annotated regions. Notably, we observed significant post-race increases of reads that correspond to repeats, especially the intergenic and intronic L1 and L2 transposable elements. We also observed increased expression of the antisense strands compared to the sense strands in intronic and regulatory regions (1 kb up- and downstream) of the genes, suggesting that antisense transcription could be one of the main mechanisms for transposon regulation in the horse under stress conditions. We identified a large number of transcripts corresponding to intergenic and intronic regions putatively associated with new transcriptional elements. Gene expression and pathway analysis allowed us to identify several biological processes and molecular functions that may be involved with exercise-induced stress. Ontology clustering reflected mechanisms that are already known to be stress activated (e.g., chemokine-type cytokines, Toll-like receptors, and kinases), as well as “nucleic acid binding” and “signal transduction activity” functions. There was also a general and transient decrease in the global rates of protein synthesis, which would be expected after strenuous global stress. In sum, our network analysis points toward the involvement of specific gene clusters in equine exercise-induced stress, including those involved in inflammation, cell signaling, and immune interactions.

## Introduction

Intense athletic performance is usually recognized as a stress factor, as is any environmental change that reduces cells and tissues viability or fitness. Indeed, prolonged bouts of strenuous exercise may temporarily depress various aspects of immune function, inducing an inflammation-like condition that reflects the intensity and duration of the exercise bout. It has been hypothesized that the physio-pathological condition that develops in athletes subjected to heavy training (i.e., overtraining syndrome) is caused by derangement of cellular immune regulation [1].

Cellular adaptation to a new homeostasis involves the modification of certain aspects of cell physiology. Stress responses are characterized by changes in gene expression, metabolism, cell cycle progression and protein homeostasis. These responses act over various time scales, ranging from post-translational effects that can provide immediate responses, to regulation of gene expression, which is essential for the slower, long-term adaptation and recovery phases.

As observed in most adaptive responses, the tight control of gene expression is coupled with fast response kinetics and controlled reversibility; this enables the cell to change its transcriptional direction within minutes in the presence of stress, and then return to its basal state after the stress source is removed. After the first few minutes of stress exposure, the transcriptional pattern of the cell undergoes major changes, including asynchronous activation of multiple genes. Thus, that strong coordination is needed for the proper regulation of gene expression under stress conditions [2].

The horse is an optimal *in vivo* model for studying the response of the genome to exercise-induced stress, due to its natural aptitude for athletic performance and the homogeneity of its genetic and environmental backgrounds. In the past decade, endurance racing has become a popular worldwide equestrian sport.

Endurance is one of the toughest disciplines that a horse can face: although horses evolved differently from humans, being the horses more adapted for travel and requiring less training than humans, the effort that the equine athlete is subjected to is comparable to that of a human marathoner. Average speeds are up to 15 km per hour and are maintained during competitions that range from 20 to 160 km.

During the years, the training and riding techniques have improved, the management of the horses has evolved, and the races are run at faster speeds. Moreover, endurance is a truly challenging discipline in which the horses must exercise for extended periods without injury, and may experience extreme weather conditions, difficult terrain, and huge altitude differences. The two main factors that can limit performance of animals in endurance races are dehydration/electrolyte imbalance and glycogen store depletion. Under race conditions, animals can experience increased oxidative and inflammatory stress [3], altered transcription of immuno-inflammatory genes, and a coordinated stress response that arises through multiple genomic mechanisms [4]–[8].

RNA-sequencing (RNA-seq) is a whole-transcriptome sequencing method that can capture the scale and complexity of organ- or tissue-specific transcriptomes, and is thus the technique of choice for investigating gene expression during complex phenomena, such as stress. Many studies have underlined the advantages of RNA-seq over other consolidated techniques such as microarrays. These include the identification of differentially expressed genes (as in microarrays), as well as the analysis of differentially expressed regulatory elements (non-coding, antisense RNAs, isoforms, miRNAs, etc.) that are being increasingly recognized as important in the post-genomic era [9], [10]. Furthermore, deep-sequencing methods such as RNA-seq can discover novel transcripts not anticipated in the design of the microarray, and also detect and quantify low-abundance transcripts below the detection threshold of microarray analysis [11].

This is particularly important because although less than 2% of the mammalian genome is protein-coding, cells put major effort into transcribing at low level numerous large nuclear sequences that were previously considered non-functional [12].

The recent use of next-generation sequencing technologies has shed new light on these transcription events and introduced a plethora of non-coding transcripts, including antisense transcripts [12]–[14]. Genome-wide studies have shown that many genes have antisense counterparts, stimulating investigations into their functional significance [15]. Indeed, up to 72% of transcripts have been demonstrated to have antisense partners in the human and mouse transcriptomes [16]. The best-known role of endogenous antisense transcripts is their ability to control gene expression via transcriptional repression, which can influence the epigenetic remodeling of neighboring genomic regions. In particular, natural antisense transcripts (NATs) are involved in controlling developmental processes, various stress adaptations, and the response to viral infection [17].

These unexpected findings are further complicating the scenario that was previously shattered by the discovery of widespread alternative splicing as a major source of RNA plasticity in space (tissues) and time (situations) [18]. A recent study demonstrated that antisense transcription not only plays regulatory roles, it can affect alternative splicing itself [14]. Among the identified mechanisms of alternative splicing, intron retention and exonization events seem to be involved in the stress response [19], [20]. It has been well documented that intron retention may be involved in the post-transcriptional regulation of gene expression in response to various stress conditions [21]. Although exonization is not categorized as a distinct splicing mechanism, it is an alternative splicing process by which new exons are acquired from intronic DNA sequences. Furthermore, recent studies have indicated that such events arise via transposable elements (TEs) in various species [22]. Thus, transposable elements and intronic sequences may be used to enrich transcriptomes with limited genomic resources in response to cellular stress or changing environmental conditions. Moreover, down-regulation of genes in response to heat shock was found to be mediated through antisense transcription driven by the binding of heat shock factors to antisense repeats [19], suggesting that antisense transcription may regulate the alternative splicing machinery.

We previously found that repeat-derived sequences are both highly and differentially expressed during physical effort in horses, hinting at complicated scenarios in the exercise-associated regulation of gene expression [5]. Furthermore, numerous human diseases have been associated with defects in the expression of alternative mRNA isoforms [23]. These previous findings emphasize the importance of thoroughly understanding transcription, RNA regulation, and the involvement of splicing in disease and stress responses [18].

One important aspect that should be studied further is the transcriptional response to physical stress in terms of time (different phases) and space (tissues exposed to the stimulus). Time-course studies will be particularly helpful in this regard, as they will allow researchers to study interactions between the organism and its environment without system-level variables.

Here, we sought to evaluate the broader context of changes in gene expression by applying strand-specific RNA-seq analysis via SOLiD technology in an experimental framework that focuses on the exercise-induced stress of endurance races in equine athletes.

## Materials and Methods

### Experimental design

To monitor the transcriptional landscape of exercise in the horse, we used a well-characterized experimental design [4], [6], [8], [24] to examine RNA expression changes in PBMCs (peripheral blood mononuclear cells) sampled at two time points: Basal (T1), with the athlete at rest; and Race (T2), immediately at the end of the race. For each time point we collected RNA from two biological replicates.

### Sample collection and RNA preparation

The studied animals were handled in accordance with proper animal welfare, with cooperation from the horse owners, their private veterinarians, and the official veterinary commission of the sampled race.

Briefly, Athlete's Private Veterinarians (APV) and FEI (Fédération Equestre Internationale) veterinarians approved by FEI regulation together with the Veterinary surgeons belonging to the race veterinary service collected the blood samples. Permission for sampling were required to the owner, to the mentioned vets and officially authorised by the President of Veterinary commission. Owners and APV required the clinical haematological examination and approved blood collection for experimental purposes.

In total, 28 ml of blood were taken from jugular vein using standard procedure and in particular:

- 1×3 ml EDTA Blood Collection Tube for full blood count (Haematocrit and White Blood Cell Count (WBC).
- 2×10 ml EDTA Blood Collection Tube for RNA extraction (used in this study)
- 1×5 ml serum collection tube for compulsory blood biochemical analysis.

No additional needles were used: blood collection tubes belonging to this study were inserted in the same needle holder used to collect blood for the compulsory analysis.

To ensure sample homogeneity, the enrolled subjects had common genetic and management backgrounds. Top-athlete Arabian horses were chosen from among the participants of the highest international endurance race category (160 km), and monitored during the 2009–2010 training season. Only subjects that passed FEI (Fédération Equestre Internationale) compulsory medical checks (pre-, during, and post-race) were considered for the study. The experiment was designed in great details and performed under very well defined and monitored environmental and medical conditions. In fact, each horse owner completed an epidemiological-anamnestic questionnaire that included training information, and all horses underwent medical examinations to exclude any disease, fever or medical treatment that could affect the immune system. Two study subjects were enrolled, both 11-year-old Arabian geldings that finished without injuries the same 160 km race. Blood samples were collected from the jugular vein at rest (Basal, T1) and at the end of the competition (Race, T2). Immediately after the collection, PBMCs were isolated using the Ficoll-Hypaque method (GE Healthcare, Pollards Wood, United Kingdom).

Total RNA was extracted using the Aurum Total RNA Fatty and Fibrous Tissue kit (Bio-Rad, Hercules, CA, USA) according to the manufacturer's instructions. Genomic DNA was eliminated by DNase treatment using the provided reagents. Successful removal of DNA contaminants was verified by the absence of PCR amplification of the MC1R gene (GenBank accession number X98012, primers as described by Rieder and colleagues [25]).

mRNA was further isolated with the GeneElute mRNA isolation kit (Sigma, St. Louis, MO, USA). The extracted mRNA was quantified using the Quant-It RNA assay (Invitrogen, Dorset, United Kingdom) and a VersaFluor fluorometer (Bio-Rad). The quality of the ribonucleic acid was checked with a microfluidic electrophoresis on the BioAnalyzer (Agilent, Santa Clara, CA, USA).

### Library preparation

Samples were prepared for ligation sequencing according to the protocol provided with the SOLiD whole transcriptome library kit (Applied Biosystems, SOLiD™ Whole Transcriptome Analysis Kit, PN 4409491 Rev E). Briefly, the samples were purified with the RiboMinus Concentration Module (Invitrogen, RiboMinus™ Concentration Module, K1550-05), subjected to RNase III digestion for 10 minutes, retrotranscribed, size-selected in acrylamide 6% TBE-Urea gels, and barcoded during final amplification. The libraries were sequenced using Applied Biosystems, SOLiD™ 4, which produced reads of 50 nucleotides.

### Bioinformatic analyses

The obtained reads were mapped to the *Equus caballus* genome (Equcab2.0, http://genome.ucsc.edu/) using the PASS program, version 1.64 [26]; the percentage identity was set to 90%, and one gap was allowed. The quality filtering parameters were set automatically by PASS. Horse gene prediction coordinates (ENSEMBL version 67) were downloaded from the UCSC web site (http://genome.ucsc.edu).

To better characterize the distribution of the reads on Equcab2.0, the genome was partitioned into several regions: the protein-coding genes, their 5′- and 3′ untranslated regions (UTRs; where available), coding sequences (CDS), introns, and 1 kb upstream and downstream of the genes (to include potential promoter and terminator) and intergenic regions. Repetitive elements were considered separately and were not included in the previous features. Moreover reads mapping on ribosomal rRNA were removed and not considered in the further analyses.

To assess if the observed distribution of reads across the genomic regions differed significantly between T1 and T2 samples, we used a binomial distribution-based test (the prop.test function of the R package). The test was performed independently on the two replicates, and, to be considered significant, we required a *p*-value<0.05 for both replicates. To be more conservative, for each comparison we choose to show only the highest *p*-values between the two replicates.

To avoid ambiguity in the partitioning process, we dealt conservatively with alternative splicing, combining the different isoforms and considering them as a single variant. The distribution of reads was calculated by counting the number of reads mapped to each region (in both the sense and antisense directions) using a custom python script. To calculate the relative read coverage, we normalized the number of reads mapped to a given region (multiplied by the read length) with respect to the total length of the region.

Splicing sites were analyzed with a custom python script that required at least two independent reads to map to the same site by at least 15 nt across the exon-exon junction. The reads were assembled using the Cufflinks suite, version 2.0.2 [27] without providing the reference annotation. The transcribed fragments (transfrags) were classified according to gene predictions performed using a home-made python script. We selected fragments that corresponded to an intergenic or intronic region for more than 80% of their lengths. For annotation, the BLAST program was used to perform similarity searches against the non-redundant nucleotide and protein databases downloaded from the NCBI ftp site (ftp://ftp.ncbi.nih.gov/blast/), and the NONCODE v3.0 database [28] for annotation of long non-coding RNA. Significant matches were those with e-values of at least 1e-5 and 1e-30 for the protein and nucleotide searches, respectively.

### Gene expression and pathway analysis

Differential expression between T1 and T2 was analyzed using the edgeR package, which can be used to estimate biological variation between replicate libraries and conduct exact tests of significance on small counts [29]. Pearson correlation between biological replicates was calculated using ‘cor’ function in R package (Figure S2 in File S1). The input was a matrix composed of 29,159 rows representing the Ensembl-annotated transcripts and 4 columns corresponding to the utilized libraries (two biological replicates each of T1 and T2). For a given sample, each cell of the matrix consisted of the number of reads that uniquely mapped to that transcript (read count was calculated using htseq-count program (http://www-huber.embl.de/users/anders/HTSeq/doc/overview.html). The analysis was performed for both sense and antisense counts, and the threshold level of statistical significance was set to <0.05 FDR (False Discovery Rate).

Gene Ontology (GO) enrichment analysis was performed using BiNGO 2.44 (Biological Networks Gene Ontology), a Cytoscape 2.8.3 plugin that allows the overrepresentation of GO terms to be analyzed in a given set of genes within a statistical framework [30]. Ensembl transcript IDs were converted to official gene names, and ambiguous assignments were manually checked and collapsed to a unique symbol. Records that were not mapped with the Ensembl annotation were converted to Uniprot AC and then to GeneID, (http://www.uniprot.org/?tab=batch&tab=mapping) allowing us to enrich the list of official gene names. The *Homo sapiens* annotation and the last available ontology in OBO format (Open Biological Ontologies, http://www.geneontology.org/) were used to perform the analysis for the three GO vocabularies. We converted the *Equus caballus* gene names to their *Homo sapiens* counterparts according to the HUGO naming system (http://www.genenames.org).

The enhanced lists of FDRs that were significant up-regulated (logFC>1; n = 579) and down-regulated (logFC<1; n = 255) were inserted into BiNGO for analysis. Ingenuity Pathways Analysis (IPA, Ingenuity® Systems, http://www.ingenuity.com) was used to identify the significantly enriched canonical pathways, and to build and analyze significantly enriched molecular interaction networks from the gene list. The list that was used for the BiNGO annotation was pasted into Ingenuity, and the target tissue was set as “Immune Cells”.

From the IPA library of canonical pathways, we identified the canonical pathways that were most significantly related to the data set. The significance of the association between the data set and a given canonical pathway was measured by: 1) the ratio of the number of genes from the data set that mapped to the pathway divided by the total number of genes in the pathway; and 2) Fisher's exact test.

To generate networks, we identified genes that showed significant differential regulation (called focus genes) and laid them over a global molecular network developed from information contained in the Ingenuity knowledge base. Networks were algorithmically generated based on the connectivity of the focus genes, and then ranked by a score that reflected the number of genes in the data set and the size of the network, and was the negative log of the *p*-value. The higher the score, the lower the probability of finding the observed data set of genes in a given network by chance. Functional analyses of the networks were used to identify the biological functions and/or diseases that were most significantly related to the genes in the network, as well as the potential toxicity and safety of the compounds associated with the genes of a given network.

## Results and Discussion

### Genomic landscape

Sequencing of the four libraries produced more than 260 million reads that were submitted in GenBank with the following BioProject id: PRJNA196393. After filtering out the low-quality reads, we aligned more than 40% of the remaining sequences to the genome, yielding a total of almost 80 million uniquely mapped reads. Mapping statistics are reported in Table 1.

**Table 1:** SOLiD sequencer throughput and alignment statistics.

Library	Produced Reads	Filtered (low quality)	Suitable Reads	Aligned Reads	% Aligned	Spliced Reads	Unique Reads	Alignments
**A T1**	85049611	19678362	65371249	25766232	39.42	1337871	22331445	36408516
**A T2**	74329898	15520649	58809249	24610147	41.85	1146753	22505061	32041310
**B T1**	55817371	8074602	47742769	20789902	43.55	1052474	17913219	29370920
**B T2**	53072584	8878519	44194065	18829992	42.61	879531	17198453	24211172

**Figure 1:**
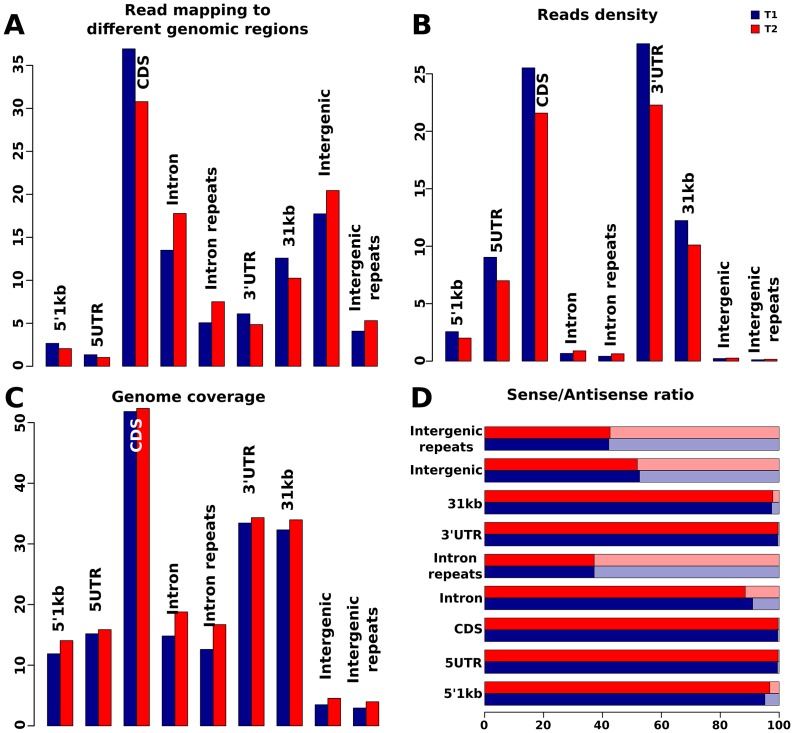
(A) Basal (T1) and race (T2) sample reads map to different genomic regions. The majority of the reads map to known genes (CDS, 3′ UTR and 5′ UTR), while a large fraction maps to non-coding regions (introns, intergenic regions, and the 1-kb regions up- and downstream of genes). Comparison between T1 and T2 show a transcriptional shift from coding to non-coding predicted regions. (B) Expression density was calculated as number of reads normalized by the lengths of each genomic region. (C) Fraction of bases covered in the different genomic regions. (D) Fraction of reads that map to the sense (light) and antisense (dark) strands in each genomic region. In the intergenic region, the fraction was calculated using the number of reads from the plus and minus strands.

We analyzed tag distribution merging the biological replicates in order to assess the contribution of the whole libraries. In addition, to verify the robustness of out results, we tested independently on the single biological replicates the significance of tag distribution differences across the genomic regions (details in material and methods). Row counts and tag distribution statistics of the single samples are provided as supplementary material (Tables S9 in File S1). The majority of the uniquely mapped reads aligned with known genes, covering more than 50% of the coding fraction (Figure 1A and B). A large portion of the reads mapped to the intronic and intergenic regions; however, when the number of reads was normalized to the total length of these regions, the relative read density was found to be very low (Figure 1 C). This was as expected, due to the high proportion of intronic versus exonic sequences in mammals. We also found that the relative tag density decreased from 1-kb upstream of the 3′ end of genes towards the 5′ end. This effect could be due to a bias of poly-A library preparation [31], casing enrichment at the 3′ end. Moreover, we noticed a higher tag density in the 1-kb region upstream of the 3′ end compared to the 3′ UTR. Since our retro-transcription strategy was based on oligo-dT priming, this finding was unexpected. We speculate that, together with the 3′ enrichment bias due to the poly-A library preparation, it may reflect incomplete prediction of the 3′ UTRs or a general inaccuracy of the gene annotations in Equcab2.0.

The basal and stressed samples had similar tag distributions (not shown), but the transcriptome profiles in the exonic and intronic regions showed substantial differences. We found a significant decrease in the fraction of reads that mapped to coding sequences in T1 versus T2 (35.5% vs. 30%; *p*-value 0.001) (Figure 1A), and a significant increase in the proportion of reads mapping to intronic regions under the stressed condition (about 1.3-fold increase, *p*-value 0.004). The intergenic regions showed a less marked increase in the frequency of mapped reads under the stressed condition, but this variation was not statistically significant. It is worth noting that the fold-change calculated within each biological replicate has an average value of 1.05, supporting the robustness of our results. All together, these results indicate that there was a shift from coding to non-coding predicted regions, in particular at intron level, suggesting that the equine response to this stress condition involves the expression regulation of new transcriptionally active regions.

**Figure 2:**
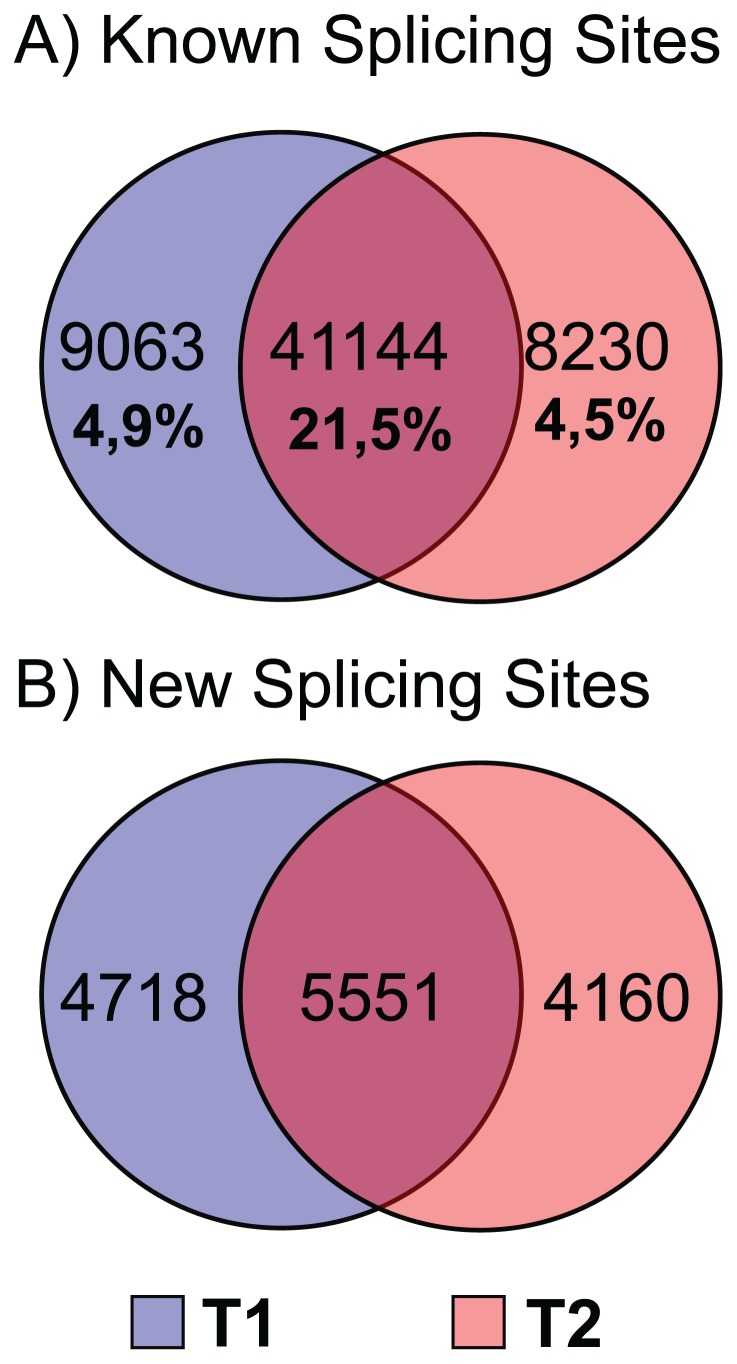
Venn diagram showing the number of splice sites identified in the T1 and T2 samples. A) Splicing sites confirmed by previously reported annotation of horse genes. B) Novel splicing sites.

One of the mechanisms that can activate new transcriptional regions is the so-called process of “exonization” or “intron retention” [32], which has been associated with the post-transcriptional regulation of gene expression in response to various stress conditions [33], [34]. These mechanisms may also be involved in the exercise-induced expression changes observed in horse. A comparison of the splicing patterns observed under the basal (T1) and stressed (T2) conditions revealed that 90% of the spliced reads from both samples aligned on known splice sites, confirming more than 30% of the predicted splicing sites in the Ensemble annotation (Figure 2A).

In addition, we identified 14,429 new splicing sites; of them, 5,551 were shared between the two conditions, 4,718 were exclusive to the T1 samples, and 4,160 were exclusive to the T2 samples (Figure 2B).

Next, we classified the observed splicing site events on the basis of the different genomic regions involved (Table 2). In both T1 and T2 samples almost half of the new splicing events occurred within coding sequences or between coding and intronic regions. Notably, there was a significant decrease (*p*-value<0.001) of “coding-coding” splicing events and a moderate increase in “coding-intron” events (*p*-value<0.05) in T2 versus T1. In particular, the percentage of intron-repeat junctions versus the total number of new splicing sites increased from 0.91 at rest to 1.27 after the race (Table 2).

**Table 2:** Splicing site events distribution of exclusive splicing sites.

New SP site (All) exlusive)		
	T1	T2
**3UTR_3UTR**	0.25	0.17
**3UTR_CDS**	0.25	0.14
**3UTR_Intergenic**	0.45	0.36
**3UTR_Intron**	0.25	0.17
**3UTR_Repeat**	0.08	0.07
**5UTR_5UTR**	0.30	0.41
**5UTR_CDS**	0.30	0.34
**5UTR_Intergenic**	1.67	1.63
**5UTR_Intron**	1.27	1.78
**5UTR_Repeat**	0.38	0.75
**CDS_CDS**	29.99	26.83
**CDS_Intergenic**	4.64	4.11
**CDS_Intron**	24.44	26.25
**CDS_Repeat**	6.04	6.54
**Intergenic_Intergenic**	17.66	18.08
**Intergenic_Intron**	0.34	0.48
**Intergenic_Repeat**	4.03	4.33
**Intron_Intron**	3.58	3.97
**Intron_Repeat**	0.91	1.27
**Repeat_Repeat**	3.16	2.33

In addition to a transcriptional shift from coding to non-coding regions, we also investigated repeats matching reads distribution. We found that the intronic repeats transcription increases from 5% to 7.5% of the total number of reads (*p*-value<0.02) in T2 compared to T1 (Figure 1A). Our results are consistent with the hypothesis that transposable elements and intronic sequences may serve as transcriptional units capable of enriching transcriptomes with limited genomic resources when necessary, such as under stress conditions [32].

Otherwise we did not observe significant shift analyzing the intergenic repeats, in which the proportion remains quite the same on both conditions.

Our investigation of the sense-antisense ratios for the different regions of the genome revealed that 99% of the reads in protein-coding regions were from the sense strand. Higher sense-antisense ratios were seen in the intronic and regulatory regions (1 kb up- and down-stream) of the genes (Figure 1D).

In contrast, the repetitive elements (e.g., members of the L1 family) showed higher levels of expression from the antisense orientation compared to the sense strand (Figure 1D), suggesting that antisense transcription is a main mechanisms for the regulation of transposons (Table 3) [35].

**Table 3:** The left part of the table shows the expression fold-change of intronic L1 and L2 transposable elements in stressed (T2) compared to the rest (T1) samples.

	Fold Change				Antisense/Sense Ratio					
	T2/T1	A2/A1	B2/B1		T1	T2	A1	B1	A2	B2
***L1***	1.56	1.62	1.49	***L1***	3.55	3.21	4.10	3.34	3.03	3.04
***L2***	1.46	1.54	1.35	***L2***	0.76	0.82	0.66	0.76	0.89	0.90
***Tot***	1.48	1.54	1.40	***Tot***	1.69	1.69	1.72	1.68	1.66	1.70

Fold-change for the single biological replicates are also reported (A1: sample A at time point 1; A2: sample A at time point 2; B1: sample B at time point 1; B2: sample B at time point 2) For each sample, the reads counts were normalized to the total number of mapped reads. In the right part of the table are indicated the results of the analysis performed considering reads aligning on both sense and antisense direction respect to the repeat strand. The third row refers to the read distribution considering the whole set of repetitive elements. The results show an higher increasing on the expression of the antisense compared to the sense strand for L1 repeat elements. On the other hand this pattern of expression is not visible for L2 members.

Otherwise, if this can be considered a general mechanism, specific classes of transposable elements show different behaviors. For example, among the most highly expressed transposable elements we found members belonging to L1 and L2 families, which account for about 33% and 17%, respectively, of the total number of repeats matching reads (Tables S9 in File S1). As shown in Table 3, both L1 and L2 elements show an increased expression in T2 compared to T1 (*p*-value of 5.6e^−7^ for L1 family and *p*-value<0.01 for L2). However, only L1 members have a higher antisense transcription (almost three fold) whereas L2 family elements show an opposite pattern of expression, suggesting different mechanisms of regulation.

When we compared the strand orientation of L1 and L2 transposable elements with the orientation of their hosting introns, we observed that 34% of L1 repeats were inserted on the same strand of the hosting intron, while 66% were inserted on the opposite strand. L2 elements showed a similar but less evident unbalance with 44% concordant with the intron strand, while 56% were inserted on the opposite strand. Pandey and colleagues [19] recently showed that repetitive elements are non-randomly distributed through the genome, and showed that these elements retain orientation-specific regulatory sites in genes related to specific biological processes. In this way, the stress-related down-regulation of a transcript could be mediated via transcription factors that bind the antisense repeats [19].

### Identification of new transcriptionally active regions

We merged the reads from all the samples and used the Cufflinks program to assemble the sequences into transcript fragments called transfrags. We obtained a total of 324,446 transfrags, most likely derived from reads that were only partially assembled due to low sequencing coverage and the use of single-end libraries.

**Figure 3:**
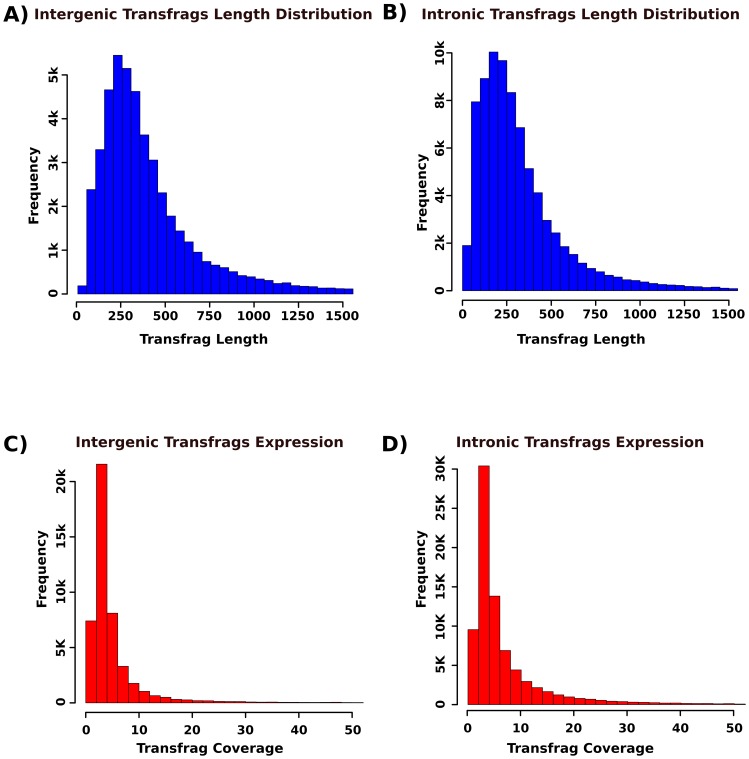
Histogram showing intergenic and intronic fragment lengths (A and B) and distribution of expression (C and D panel).

Many studies have found that the mammalian genome is extensively transcribed, giving rise to thousands of non-coding transcripts [9], [10]. It is unclear whether all of these transcripts are functional, but it is evident that there are many functional non-coding RNAs (ncRNAs) [36]. In an effort to identify new putative transcriptional elements, we focused on the transfrags that localized to intergenic and intronic regions. In the intergenic regions, we identified 47,194 transcribed elements with an average sequence length of 326 bp, while the intronic regions yielded 80,184 new transcripts with an average length of 260 bp (Figure 3A and B). For each fragment, we calculated the expression value (the number of reads normalized with respect to the transcript length). We found that most were expressed at very low levels (Figure 3C and D).

We annotated these novel transcripts by performing similarity searches against the non-redundant protein database and non-redundant nucleotide database of the NCBI, and the NONCODE V3 database (a collection of non-coding transcripts). Only 17.8% of the intronic transcripts matched similar sequences in at least one of the three databases, while 25.8% of the intergenic transfrags had matches. The transcripts that showed similarities to known proteins most likely represented genes or exons that our analysis missed due to incomplete gene prediction, or newly annotated genes outside the official Ensembl release (Table S1 in File S1). Conversely, we identified significant similarities for numerous sequences at the nucleotide level, especially to BAC sequences and genomic regions of other mammalian organisms, suggesting that the majority of the transcripts are non-coding (Table 3). In addition, we found 779 intronic and 1,249 intergenic transcripts that yielded significant similarities with sequences in the NONCODE database (Table 4).

**Table 4:** Annotation results according to cufflinks intronic and intergenic fragments output.

Annotation		
	Intron	Intergenic
**NONCODE db**	779	1249
**NT**	13473	11389
**NR**	2765	3208

The transcripts were search against the non redundant nucleotide database (NT), non redundant protein database (NR) and a database of non coding sequences (NONCODE).

The complexity of the mammalian transcriptional landscape is well known, and recent high-throughput transcriptomic analyses have revealed that eukaryotic genomes undergo transcription of up to 74% of their genomic DNA [9]. The functional significance of these transcripts is still controversial; it has been reported that more than 95% show little evidence of evolutionary conservation and are expressed at extremely low levels. These transcripts were previously thought to be just transcriptional noise, with no function [37], [38]. However, emerging evidence indicates that they represent novel classes of non-coding RNAs that can be very tissue-specific and may influence transcription through a variety of mechanisms [10], [39].

The identification and annotation of non-coding RNAs (ncRNAs) is quite complex and requires integration of the coding potential of the RNA and the chromatin modifications of the corresponding genomic region. As it can be challenging to determine if transcripts are non-coding and whether they have biological function, we focused on transcripts that showed differential expression before and after the stress event. Such differential expression is likely to reflect genuinely functional transcripts. We identified 167 intergenic transcripts that were down-regulated and 635 that were up-regulated at T2 versusT1. Similarly, we found 177 intronic transcripts that were down-regulated versus 705 that were up-regulated in the stressed sample. The large majority of these transcripts did not have any homologous sequence in the databases. Interestingly, however, 30 intronic transcripts and 39 intergenic transcripts showed significant similarities with known long non-coding RNA (lncRNA) sequences (Table S2 in file S1, Sequences S1 in file S2, and Sequences S2 in file S3).

Many studies have found that lncRNAs can critically affect gene expression or guide chromatin-modifying complexes to specific genomic loci, thereby establishing cell-type-specific epigenetic states [36], [40], [41]. These findings suggest that ncRNAs, particularly lncRNAs, may play important roles in regulating gene expression in response to stress.

### Gene expression and pathway analysis

A transcriptome comparison of the two samples was performed in order to test the variation between horses. We calculated the Pearson correlation (Figure S2 in File S1) between the samples with the aim of assaying the extent of similarity of the gene expression profile of the two horses. We found that the correlation is as high as 0.97 at time T1 and 0.94 at time T2, demonstrating that the two horses give rise to very similar profiles and they behave similarly in response to stress. This finding confirms that our experimental data are robust and consistent with the gene expression changes that occur between animals at rest and after competition.

Our edgeR-based analysis of differential gene expression between T1 and T2 identified 1,154 differentially expressed transcripts from the sense counts: 579 up-regulated [with a log fold-change (logFC)>1] and 255 down-regulated (logFC<−1). From the antisense counts, we identified 16 differentially expressed transcripts: 11 that were up-regulated and 5 that were down-regulated. Complete lists of significant FDR transcripts (both sense and antisense) can be found in the Supplementary Materials (Table S3 in File S1). Top genes like (*IL8*, *IL18*, *MMP1*, *CCL5* etc.) were found to be differentially expressed between the two conditions confirming previous results on the same experimental design with other subjects [4].

We analyzed the above-described (material and method section) enriched list with the Biological Networks Gene Ontology (BiNGO) program to identify the ontologies involved in the gene networks, including all the three Gene Ontology (GO) vocabularies (cellular components, molecular functions and biological processes). The BiNGO analysis identified several biological processes that may be involved with exercise-induced stress in the equine athlete. The up-regulated processes included the “response to stress” as the most significant node (146 of the 457 annotated genes), followed by the “defense response,” “response to stimulus” and “response to wandering”. The down-regulated biological processes included “positive regulation of biological processes,” “regulation of immune system processes” and “positive regulation of cellular processes” (Table S4 in File S1).

The results of our ontology clustering reflected some well-known stress-activated factors, including chemokines (small secreted cytokines), Toll-like receptors (which play key roles in the innate immune system), and protein kinases. Consistent with these results, our analysis of ontology revealed that the most significantly up-regulated functions were “nucleic acid binding” and “signal transduction activity.” In response to severe stress, we would expect to see a transient decrease in the production of growth-related proteins and a general decrease in global protein synthesis [2]. Indeed, we observed this pattern in the down-regulated biological processes and molecular function ontologies (Table S4 in File S1).

We further used Ingenuity Pathway Analysis (IPA) to identify the biological mechanisms, pathways and functions most relevant to the 1,154 putative differentially expressed genes. A total of 1,001 genes were mapped and included in the analysis, whereas the remaining 153 unmapped genes were excluded. The results are summarized in Table 5. The analysis comprised 10 networks (Table S5 in File S1); the two most significant networks were identified as Network 1, “Cellular Function and Maintenance, Cell-To-Cell Signaling and Interaction, Hematological System Development and Function” (score 76, focus molecules 70/70), and Network 2, “Inflammatory Response, Cell-To-Cell Signaling and Interaction, Hematological System Development and Function” (score 27, focus molecules 43/70). The two most important bio-functions for the category “Diseases and Disorders” were “inflammatory response” (1.54E-20<*p*-value<1.98E-02) with 154 molecules, and “inflammatory diseases” (8.92E-13<*p*-value<1.39E-02) with 59 molecules. The most important bio-function for the category “Molecular and Cellular Functions” was “cell-to-cell signaling and interaction” (1.54E-20<*p*-value<1.59E-02) with 137 molecules (Table 5).

**Table 5:** IPA networks summary results.

Associated Network Functions	
*Name*	*Score*
Cellular Function and Maintenance, Cell-To-Cell Signaling and Interaction, Hematological System Development and Function	76
Inflammatory Response, Cell-To-Cell Signaling and Interaction, Hematological System Development and Function	27
Cellular Movement, Hematological System Development and Function, Immune Cell Trafficking	26
Cell-To-Cell Signaling and Interaction, Infectious Disease, Hematological System Development and Function	21
Cell-To-Cell Signaling and Interaction, Hematological System Development and Function, Immune Cell Trafficking	20

Diseases and disorders		
*Name*	*p-value*	*# of Molecules*
Inflammatory Response	6.69E-21 - 2.06E-02	154
Infectious Disease	1.45E-15 - 1.46E-02	49
Respiratory Disease	1.45E-15 - 1.45E-15	31
Connective Tissue Disorders	1.00E-12 - 1.26E-03	47
Inflammatory Disease	1.00E-12 - 1.41E-02	59

Molecular and Cellular Functions		
*Name*	*p-value*	*# of Molecules*
Cell-To-Cell Signaling and Interaction	6.69E-21 - 2.06E-02	137
Cell Death and Survival	1.01E-17 - 1.69E-02	91
Cellular Function and Maintenance	1.79E-16 - 1.69E-02	118
Cellular Development	2.59E-14 - 2.01E-02	127
Cellular Growth and Proliferation	2.59E-14 - 1.69E-02	100

Physiological System Development and Function		
*Name*	*p-value*	*# of Molecules*
Hematological System Development and Function	6.69E-21 - 2.06E-02	203
Immune Cell Trafficking	6.69E-21 - 2.06E-02	149
Tissue Morphology	1.63E-15 - 1.47E-02	109
Hematopoiesis	1.22E-11 - 2.01E-02	91
Tissue Development	4.43E-11 - 2.06E-02	83

Molecules	
*Name*	*Expression Value (log10)*
IL22RA2	8.907
PDK4	6.544
MARCO	5.334
MMP27	5.038
MMP8	5.026
RETN	4.862
IL1R2	4.753
KMO	4.481
COL4A1	4.178
PGLYRP1	4.117
FLT4	−6.645
CD34	−5.711
EEF1A2	−4.506
BMP2	−4.315
DAPK2	−4.149
GATA2	−3.88
GZMB	−3.428
GPR56	−3.077
PTPRO	−2.936
CD7	−2.901

The networks and bio-functions reflect two well-described phenomena observed during exercise-induced stress: inflammation and immune cell signaling [42], [43].

**Figure 4:**
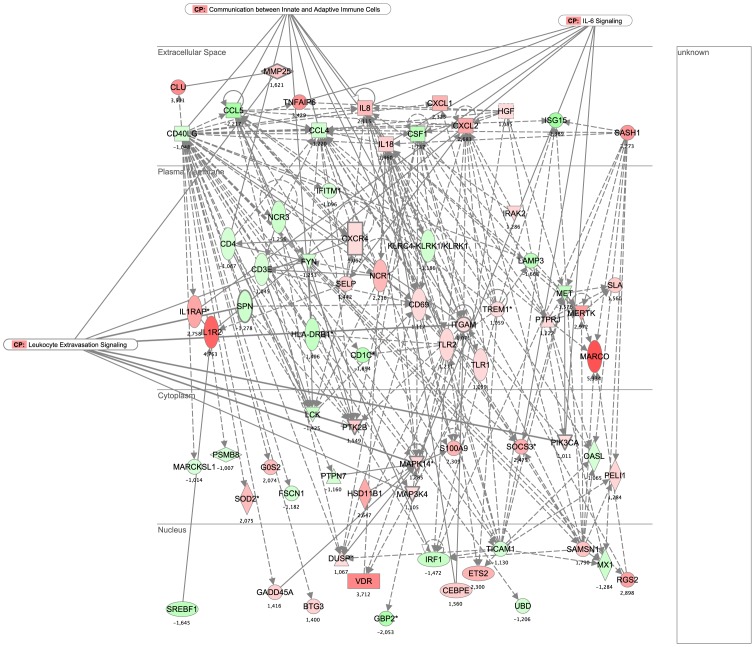
Network 1 and 2 results from IPA analysis. Genes or gene products are represented as nodes, and the biological relationship between two nodes is represented as an edge. All connections are supported by at least one reference from the literature or canonical information stored in the Ingenuity knowledge base. The intensity of the node color indicates the degree of up-regulation (red) or down-regulation (green).

Network 1, which was the highest ranked network, was composed of genes related to immune responses, cell defense, cell cycle processes, and the movement, trafficking and differentiation of immune cells. The pro-inflammatory actions of various chemokines and Toll-like receptor signaling pathways play a central role and appear to interact with critical cell cycle regulators (Figure 4).

**Figure 5:**
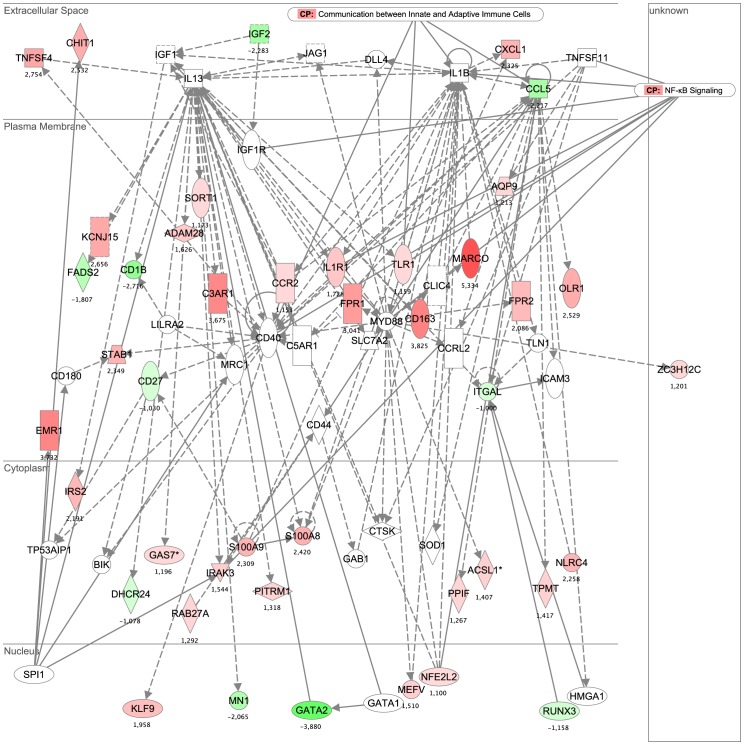
Network 1 and 2 results from IPA analysis. Genes or gene products are represented as nodes, and the biological relationship between two nodes is represented as an edge. All connections are supported by at least one reference from the literature or canonical information stored in the Ingenuity knowledge base. The intensity of the node color indicates the degree of up-regulation (red) or down-regulation (green).

The genes of Network 2 were associated with inflammatory responses, cellular movement, and immunological disease. These include pro-inflammatory factors related to the NF-kB pathway (e.g., CXCL1, IL1R1, CCR2, TLR1, CCL5) and molecules necessary for the extravasation of leukocytes during inflammation (e.g., ITGAL and MARCO) (Figure 5). When we superimposed “diseases and functions” on the merged cores of Networks 1 and 2 in IPA, we obtained 101 genes associated with the “inflammatory response” (1.54E-42<*p*-value<2.49E-01) and 86 genes associated with “cell-to-cell trafficking” (1.54E-42<*p*-value<2.49E-01). The most significantly represented canonical pathway (2.05E-4), which was associated with Network 1, was “communication between innate and adaptive immune cells”. This highlights the importance of different modes of cross-talk between the innate and adaptive immune systems, such as via soluble factors (chemokines and cytokines) and cell-to-cell communication (Figure S1 in File S1).

The immune response to an infection or other stress (i.e., physical stress) can be divided into the innate and adaptive phases. Activation of the innate immune system results in rapid action, whereas the adaptive immune response develops after a time lag, but results in the activation of cells that are highly specific. Our experimental findings fit within this framework, as the response to exercise is known to mimic the response to various other stresses, wherein the immune system attempts to defend the organism against destructive forces [4], [8], [44], [45]. Various molecules involved in this pathway were found in Networks 1 and 2 and were all strongly modulated by stress in our system; these include the down-regulated factors, CCL4, CCL5, CD4 and CD8, and the up-regulated factors, IL8, IL18 and TLR (Table S6 in File S1).

Another canonical pathway that showed a low *p*-value (2.34E-04) and is known to be involved in exercise-induced stress is “leukocyte extravasation signaling,” which is the process by which leukocytes migrate from blood to tissues during inflammation. Strenuous exercise leads to a transient inflammatory status in human athletes [46]–[49] and model animals [50], [51], including the horse [4], [7], [8]. This pathway involves molecules for the capture, adhesion and transmigration of leukocytes. As many as 21 of the genes found to be strongly modulated in our data set encode proteins related to this transient inflammatory status, including *CXCR4*, integrins (*ITGAL* and *ITGAM*), kinases (*MAP3K4* and *MAPK14*) and metalloproteinases (*MMP1*, *-8*, *-25*, and *-27*) (Table S7 in File S1).

Superimposition of the canonical pathways with Network 1 showed that the IL6 signaling pathway is involved, with eight highly up-regulated molecules (Figure 4). Interleukin 6 (IL6) is a regulator of acute-phase responses, a lymphocyte-stimulating factor and a central chemokine in exercise-response modulation [52]–[54]. Our data set revealed the up-regulation of many IL6-type cytokines, including IL8, IL18, IL1 receptors, and the inducible negative regulator of cytokine signaling, SOCS3. IL6 also activates the mitogen activated protein kinase (MAPK) pathway, which was represented here by the up-regulations of MAPK4 and MAPK14 (Table S8 in File S1).

The most highly up-regulated and down-regulated genes identified by our IPA analysis (Table 5) were all consistent with the phenomena of stress and inflammation. The most highly up-regulated gene was *IL22A2*, which encodes IL22 binding protein (IL22BP; a soluble receptor of IL22). The true biological relevance of these soluble cytokine receptors remains unclear. One possibility is that they could function as neutralizing agents by blocking cytokines. Another possibility is that they could serve as carrier proteins, prolonging the half lives of cytokines and enhancing long-distance signaling [55]. IL22 and its receptors have been implicated in several chronic inflammatory diseases, and IL22RA2, which regulates shared central pathways, is a common risk gene for several immune disorders [56]. Additional studies will be required to fully characterize these disease-related functions. Based on our observation that *IL22A2* was the top up-regulated gene, we hypothesize that it acts via immune deregulation (phenomenon known as overreaching and overtraining in athletes) in race-stressed horses. [1], [57]. Finally, the most highly down-regulated genes were related to protein synthesis (EEF1A2), growth factors, signal transducers, and cell cycle regulators (GATA2, BMP2, GPR56, FLT4), all of which are known to be suppressed during severe stress [2].

## Conclusions

Monitoring of the transcriptional landscape before and after physical stress in equine athletes revealed differential expression of numerous genes related to inflammation and immune system activation, along with a sharp shift in expression from coding to non-coding transcripts. The latter finding suggests that the stress response involves the regulation and activation of new and uncharacterized transcriptionally active regions.

Together with the post-stress increases in the expression levels of repeats (L1 and L2) and antisense strands compared to the sense strands, both in intronic and regulatory regions, our results suggest that repeat-driven exonization may be a stress response in the horse, and that antisense transcription could be one of the main mechanism of transposon regulation. Although preliminary, our experimental data contribute to our detailed understanding of the complex transcriptional changes that occur following physical stress in equine athletes. In the future, similar results in additional horses and other livestock species may facilitate the inclusion of stress response potential in selection plans, allowing breeders to choose more stress-resistant subjects in the hopes of improving performance and preserving animal welfare. Moreover, an improved understanding of how and when remodeling of the genome structure modifies the transcriptome in response to stresses may facilitate the diagnosis of inflammatory-related diseases (e.g., autoimmune issues, cancer, etc.) and certain overtraining syndromes in human athletes.

## Supporting Information

File S1 **Figure S1**. Canonical pathway enrichment from IPA analysis. Red and green are respectively up-regulated and down-regulated fraction of the genes of the dataset. Pathways are ordered in −log(p-value) –wise form. **Figure S2**. Scatterplot of the logarithmic reads count between the samples. Pearson correlation coefficient (r) is reported for each comparison. Labels A and B represent the samples (biological replicates), while labels 1 and 2 represent the time points (at rest and after the competition). **Table S1**. Similarities of new transcripts with known proteins. **Table S2**. Transcripts with significant similarities with known long non-coding RNA. **Table S3**. Complete list of significantly modulated transcripts. **Table S4**. BiNGO analysis results. **Table S5**. Network list in the IPA analysis. **Table S6**. Canonical pathway “communication between innate and adaptive immune cells”. **Table S7**. Canonical pathway “leukocyte extravasation signaling”. **Table S8**. Canonical pathway “mitogen activated protein kinase (MAPK)”. **Table S9**. Row counts and tag distribution statistics of the single samples.
(ZIP)

File S2 **Sequences_S1.fasta.** Fasta file containing non coding sequences falling in intergenic regions.
(ZIP)

File S3 **Sequences_S2.fasta.** Fasta file containing non coding sequences falling in intronic regions.
(ZIP)
