# Supplementary figures and images for: RNA Sequencing of the Exercise Transcriptome in Equine Athletes
Source: PLoS One. 2013 Dec 31;8(12):e83504. doi: 10.1371/journal.pone.0083504 (PMC3877044; doi:10.1371/journal.pone.0083504)

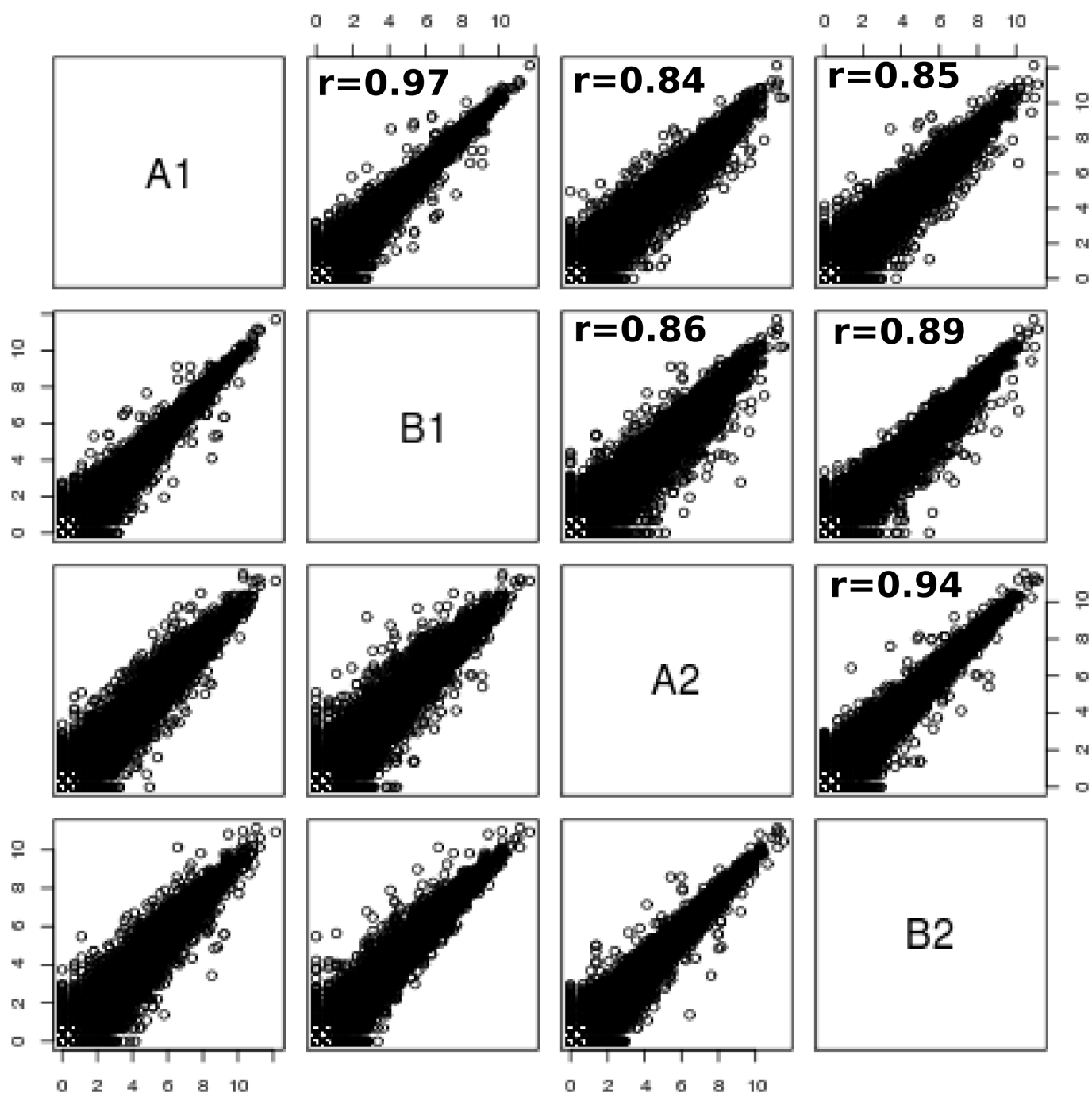

Supplement: File S1 — Figure S1. Canonical pathway enrichment from IPA analysis. Red and green are respectively up-regulated and down-regulated fraction of the genes of the dataset. Pathways are ordered in −log(p-value) –wise form. Figure S2. Scatterplot of the logarithmic reads count between the samples. Pearson correlation coefficient (r) is reported for each comparison. Labels A and B represent the samples (biological replicates), while labels 1 and 2 represent the time points (at rest and after the competition). Table S1. Similarities of new transcripts with known proteins. Table S2. Transcripts with significant similarities with known long non-coding RNA. Table S3. Complete list of significantly modulated transcripts. Table S4. BiNGO analysis results. Table S5. Network list in the IPA analysis. Table S6. Canonical pathway “communication between innate and adaptive immune cells”. Table S7. Canonical pathway “leukocyte extravasation signaling”. Table S8. Canonical pathway “mitogen activated protein kinase (MAPK)”. Table S9. Row counts and tag distribution statistics of the single samples. (ZIP) [file pone.0083504.s001.zip › Supp_Materials/Figure_S2.pdf]
