# Supplementary material for: RNA Sequencing of the Exercise Transcriptome in Equine Athletes
Source: PLoS One. 2013 Dec 31;8(12):e83504. doi: 10.1371/journal.pone.0083504 (PMC3877044; doi:10.1371/journal.pone.0083504)
Supplement: File S1 — Figure S1. Canonical pathway enrichment from IPA analysis. Red and green are respectively up-regulated and down-regulated fraction of the genes of the dataset. Pathways are ordered in −log(p-value) –wise form. Figure S2. Scatterplot of the logarithmic reads count between the samples. Pearson correlation coefficient (r) is reported for each comparison. Labels A and B represent the samples (biological replicates), while labels 1 and 2 represent the time points (at rest and after the competition). Table S1. Similarities of new transcripts with known proteins. Table S2. Transcripts with significant similarities with known long non-coding RNA. Table S3. Complete list of significantly modulated transcripts. Table S4. BiNGO analysis results. Table S5. Network list in the IPA analysis. Table S6. Canonical pathway “communication between innate and adaptive immune cells”. Table S7. Canonical pathway “leukocyte extravasation signaling”. Table S8. Canonical pathway “mitogen activated protein kinase (MAPK)”. Table S9. Row counts and tag distribution statistics of the single samples. (ZIP) [file Figure_S1.pdf › Supp_Materials/Figure_S1.pdf]

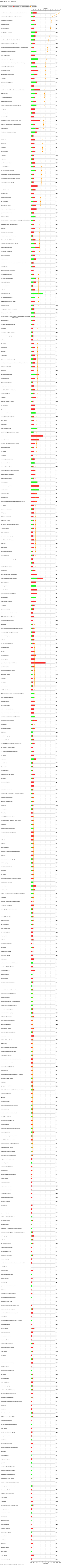
Analysis: Mappati +1 -1 Immune cells
Downregulated
No change
Upregulated
No overlap with dataset
-log(p-value)
Percentage
0 10 20 30 40 50 60 70 80 90 100 110 120
Role of Pattern Recognition Receptors in Recognition of Bacteria and Viruses 90
Communication between Innate and Adaptive Immune Cells 81
Leukocyte Extravasation Signaling 176
T Cell Receptor Signaling 96
Atherosclerosis Signaling 98
PKCθ Signaling in T Lymphocytes 111
Type I Diabetes Mellitus Signaling 101
Crosstalk between Dendritic Cells and Natural Killer Cells 82
Hepatic Fibrosis / Hepatic Stellate Cell Activation 120
Role of Macrophages, Fibroblasts and Endothelial Cells in Rheumatoid Arthritis 268
Hematopoiesis from Pluripotent Stem Cells 44
Toll-like Receptor Signaling 55
Calcium-induced T Lymphocyte Apoptosis 56
Production of Nitric Oxide and Reactive Oxygen Species in Macrophages 159
Autoimmune Thyroid Disease Signaling 39
Role of IL-17A in Psoriasis 13
NAD biosynthesis II (from tryptophan) 15
IL-6 Signaling 108
Nur77 Signaling in T Lymphocytes 50
LXR/RXR Activation 98
Tryptophan Degradation to 2-amino-3-carboxymuconate Semialdehyde 8
Interferon Signaling 31
CCR5 Signaling in Macrophages 65
Semaphorin Signaling in Neurons 42
Cytotoxic T Lymphocyte-mediated Apoptosis of Target Cells 53
IL-10 Signaling 65
Chemokine Signaling 65
Eicosanoid Signaling 59
Airway Pathology in Chronic Obstructive Pulmonary Disease 8
Altered T Cell and B Cell Signaling in Rheumatoid Arthritis 79
Granzyme B Signaling 16
CTLA4 Signaling in Cytotoxic T Lymphocytes 81
Hepatic Cholestasis 121
TREM1 Signaling 58
HIF1α Signaling 95
HGF Signaling 96
Complement System 27
NF-κB Signaling 154
IL-8 Signaling 170
Role of Tissue Factor in Cancer 100
Natural Killer Cell Signaling 101
iCOS-iCOSL Signaling in T Helper Cells 101
D-myo-inositol (1,4,5,6)-Tetrakisphosphate Biosynthesis 102
D-myo-inositol (3,4,5,6)-tetrakisphosphate Biosynthesis 102
G-Protein Coupled Receptor Signaling 217
Guanosine Nucleotides Degradation III 11
Role of IL-17A in Arthritis 53
p38 MAPK Signaling 105
Renal Cell Carcinoma Signaling 67
Fatty Acid α-oxidation 12
MSP-RON Signaling Pathway 44
Mitochondrial L-carnitine Shuttle Pathway 13
3-phosphoinositide Biosynthesis 127
OX40 Signaling Pathway 58
Differential Regulation of Cytokine Production in Intestinal Epithelial Cells by IL-17A and IL-17F 23
Superpathway of Inositol Phosphate Compounds 157
p53 Signaling 85
Inhibition of Matrix Metalloproteases 35
LPS/IL-1 Mediated Inhibition of RXR Function 173
γ-linolenate Biosynthesis II (Animals) 16
Tumoricidal Function of Hepatic Natural Killer Cells 24
Superoxide Radicals Degradation 6
IL-17A Signaling in Gastric Cells 25
Macropinocytosis Signaling 62
Granzyme A Signaling 15
Role of Osteoblasts, Osteoclasts and Chondrocytes in Rheumatoid Arthritis 193
SAPK/JNK Signaling 90
NRF2-mediated Oxidative Stress Response 164
CD27 Signaling in Lymphocytes 51
Graft-versus-Host Disease Signaling 39
T Helper Cell Differentiation 65
cAMP-mediated signaling 184
IL-2 Signaling 52
Melatonin Degradation III 2
Lipid Antigen Presentation by CD1 17
Purine Nucleotides Degradation II (Aerobic) 17
Dendritic Cell Maturation 156
IL-12 Signaling and Production in Macrophages 111
CD28 Signaling in T Helper Cells 111
